# Heart rate variability (HRV) in adolescent females with anxiety disorders and major depressive disorder

**DOI:** 10.1111/j.1651-2227.2009.01657.x

**Published:** 2010-04

**Authors:** E Henje Blom, EM Olsson, E Serlachius, M Ericson, M Ingvar

**Affiliations:** 1 Department of Clinical Neuroscience, and Osher Center for Integrative Medicine, Karolinska InstitutetStockholm, Sweden; 2 Department of Psychology, Uppsala UniversityUppsala, Sweden; 3 Department of Woman and Child Health, Karolinska InstitutetStockholm, Sweden; 4 Swedish National Defence CollegeStockholm, Sweden

**Keywords:** Adolescent females, Anxiety disorders, Autonomic regulation, Heart rate variability, Major depressive disorder, Selective serotonin reuptake inhibitors

## Abstract

**Aim:**

The aim of this study was to investigate heart rate variability (HRV) in a clinical sample of female adolescents with anxiety disorders (AD) and/or major depressive disorder (MDD) compared with healthy controls and to assess the effect of selective serotonin reuptake inhibitors (SSRI) on HRV.

**Methods:**

Heart rate variability was measured in adolescent female psychiatric patients with AD and/or MDD (n = 69), mean age 16.8 years (range: 14.5–18.4), from 13 out-patient clinics and in healthy controls (n = 65), mean age 16.5 years (range: 15.9–17.7). HRV was registered in the sitting position during 4 min with no interventions.

**Results:**

Logarithmically transformed high frequency HRV (HF), low frequency HRV (LF) and standard deviation of inter beat intervals (SDNN) were lower in the clinical sample compared with the controls (Cohen’s *d* for HF = 0.57, LF = 0.55, SDNN = 0.60). This was not explained by body mass index, blood pressure or physical activity. Medication with SSRI explained 15.5% of the total variance of HF, 3.0% of LF and 6.5% of SDNN.

**Conclusions:**

Adolescent female psychiatric patients with AD and/or MDD show reduced HRV compared with healthy controls. Medication with SSRI explained a part of this difference.

## Introduction

The prevalence of self-reported symptoms of anxiety in adolescents has increased more than threefold during the last two decades in Sweden, reaching 33.1% in females and 11.9% in males (1). International studies show great discrepancies in the prevalence of anxiety disorders (AD), with 1-year prevalence ranging 2.8–18.4% in 15–18 year olds. The lifetime prevalence of major depressive disorder (MDD) of around 20% at the end of adolescence (2).

Adolescents show high correlations between self-report measures of anxiety and depression and high comorbidity rates of AD and MDD. AD tend to precede the onset of depressive disorders and late adolescents show higher comorbidity rates than younger children.

The high comorbidity rates may be partly explained by overlapping items and poor discriminant validity between depression and anxiety assessment scales that could lead to an overestimation of the association of the two constructs (3).

Clarke and Watson (4) proposed that anxiety and depression share a common component of negative affect (NA) and that NA accounts for symptom overlap and comorbidity. NA is a general factor of subjective distress and encompasses mood states such as fear, sadness, anger, guilt, scorn and disgust. They further suggested that anxiety and depression can be differentiated by increased physiological hyperarousal (autonomic hyperactivity and motor tension) specifically in anxiety and low positive affect (PA) specifically for depression. PA includes feelings of joy, energy, enthusiasm, interest, alertness and self-confidence and low PA indicates anhedonia (4). Longitudinal data confirm that anxiety- and depressive disorders are caused by a combination of shared and disorder-specific factors in adults (5). Adult measures of tripartite constructs have been modified for children and adolescents, and the concurrent and discriminant validity of anxiety and depression in youth has been investigated and new assessment scales developed (6).

Heart rate variability (HRV) has been used to test whether hyperarousal is specific for anxiety. HRV provides a window onto autonomic regulation of the heart. High frequency of HRV (HF) is related to vagal activity and includes the respiratory sinus arrhythmia (RSA) when the breathing rate is normal. Low HF has been interpreted as a sign of hyperarousal. Recent studies suggest that also low frequency HRV (LF) mainly reflects vagal influence (7). Evidence for an association between decreased vagal tone and AD (8,9), and by evidence for unchanged vagal tone in patients with MDD (10) support Watson’s model. However, decreased vagal tone in HRV measures have also been demonstrated in patients with depression (11) contradicting the model.

It has been debated whether the findings of the decreased HRV in patients with MDD is explained by comorbidity of AD (12) and/or decreased physical activity (13). Recent studies of large samples show that medication with Selective Serotonin Reuptake Inhibitors (SSRI) contributes to the decrease of HRV in patients with both MDD (14) and AD (15).

A large population based study of children aged 10–13 years, showed more complex and gender dependent relationships between HRV and measures of anxiety and depression than previously presented (16). A female predominance of mood disorders occurs in the developmental transition in mid puberty. Changes in androgen and oestrogen levels have been proposed as the causal factors (17) and may also influence HRV.

One of the most prominent confounding factors, when studying the relationship between HRV and AD and/or MDD is probably physical activity. Physical activity is associated with increase of HRV in adults (18) and predicts HRV in healthy adolescents (19). Other possible confounding factors in this context are cardiovascular risk factors, as HRV is altered among patients with ischaemic heart disease when compared with their age-matched controls (20). Already in adolescence, increased body mass index (BMI) (21), hypertension (22) and hyperglycaemia (23) are associated with decreased HRV. Thus, in a study designed to investigate the relationship between HRV and AD/MDD these parameters must be controlled for.

This study aimed to investigate HRV in female adolescent psychiatric patients with AD, MDD and comorbidity of AD/MDD compared with age matched controls, after adjustment for cardiovascular risk factors such as BMI, systolic (SBP) and diastolic blood pressure (DBP), p-glucose and physical activity. The impact of SSRI on HRV was also assessed.

## Methods

The clinical sample consisted of adolescent girls (n = 69) with a mean age 16.8 years (range: 14.5–18.4 years) who were psychiatric patients and had a Development and Wellbeing Assessment (DAWBA) validated diagnosis of one or several AD (general anxiety disorder, social phobia, specific phobia, panic disorder, separation anxiety, post-traumatic stress disorder) and/or MDD. The subjects had ongoing treatment contact (median duration 11 months) at one of 13 open psychiatric clinics for children and adolescents situated in the centre of Stockholm, surrounding suburbs or in smaller towns nearby. The staff at the clinics (psychiatrists, psychologists and social workers) asked their patients about participation and gave them written information. From what has been reported from the staff, 85% of the informed patients participated. The reason not to participate was fear of blood sampling.

**Figure 1:**
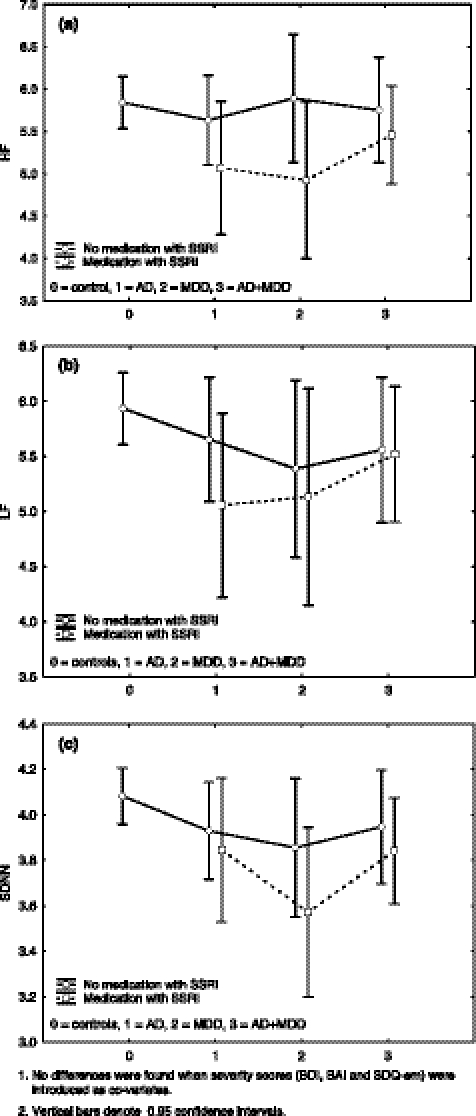
Least square means of HF (a), LF (b) and SDNN (c) in the controls and the diagnostic subgroups of anxiety disorders (AD), major depressive disorder (MDD) and comorbidity of AD/MDD with or without selective serotonin reuptake inhibitors medication. Adjustment was carried out for BMI, systolic and diastolic blood pressure, p-glucose and physical activity.

Psychiatric assessment by child and adolescent psychiatrists or psychologist and DAWBA-interview were used to establish case status of AD and/or MDD as the major diagnosis/es. Patients with severe autism or psychotic symptoms were not recruited to the study. The DAWBA interviewers worked from computerized versions of the test in which all information had been collated by software. Two of the authors independently rated the computer-generated information to decide whether or not to accept the computer diagnoses. Six subjects were denied participation because the DAWBA was incomplete or could not confirm diagnose of AD and/or MDD (Fig. 1). The measurements were performed at the patient’s clinic by the first author and an assistant and followed the same order in all subjects.

The controls consisted of adolescent girls (n = 66), with a mean age of 16.5 years (range: 15.9–17.7 years). Oral and written information about the study was given to the students in their classrooms. About 80% of the informed students participated, the participation ratio being similar across all schools. The main reasons not to participate were fear of blood sampling and reluctance to miss school-hours.

The clinical sample and the controls were age matched and recruited from similar areas. Exclusion criteria for both samples were diabetes, thyroid dysfunction, pregnancy and more than 5% missing or distorted data in any registered HRV segment. The study was approved by the Regional and Central Ethic’s Committee at Karolinska Institutet.

### Heart rate variability – measurement and processing

The subjects were sitting upright in silence with spontaneous breathing. No body movements were allowed during the procedure. None of the subjects had clinical signs or symptoms of infectious disease. Use of tobacco (snuff and smoking of cigarettes) or intake of tea, coffee, caffeine containing soft drinks or beta stimulant asthma medication was not allowed 1 h prior to the measurements. The HRV registration was preceded by 15 min of rest. HRV was measured during 2 min × 2, with blood pressure checked in between. This was a modified version of a 12 min protocol (24). The mean of the two periods for HF; LF and SDNN was calculated and then logarithmically transformed. The 2 min × 2 HRV registration has previously been shown to generate stable results in repeated measures 6 month later in the non-clinical sample, supporting that short registrations without standardized interventions can capture HRV differences of possible predictive and clinical value (19).

The square root of variance, i.e. the standard deviation of the interbeat intervals (SDNN), was used as a time domain measure reflecting all the cyclic components responsible for variability in the period of recording. In spectral analyses the variability distributes as a function of frequency. The HF (0.15–0.4 Hz) and LF (0.04–0.15 Hz) were used as frequency domain measures. The equipment was a I-330-C-2 Physiological Monitoring System (J&J Engineering; Poulsbo, WA, USA), and c-Stress customized software (PBM Systems, Stockholm, Sweden). The electrocardiogram (ECG) was recorded from electrodes placed on the left and right wrist with a sampling rate of 1024 Hz. Interbeat intervals were calculated on-line using an R-wave peak detection algorithm and stored on a PC for off-line processing. Fourier analysis was performed on 2-min segments of detrended data passed through a Hamming window. The HRV mean values were logarithmically transformed. Inter-beat-intervals were scanned manually for ectopic beats and artefacts, which were replaced with cubic spline interpolation.

### Psychiatric self-assessment scales and interviews

Development and Wellbeing Assessment (DAWBA) is a semi-structured diagnostic interview designed to generate ICD-10 and DSM-IV psychiatric diagnoses on 5–17 year olds. DAWBA has consistently generated sensible estimates of prevalence and association with risk factors. When compared with clinical diagnoses, DAWBA diagnoses support good validity (25). In this study, the information was only collected from the patients and not from parents and teachers.

Beck’s Depression Inventory (BDI) is a frequently used rating scale in adolescent psychiatry for screening and measuring symptoms of depression over a 2 week period prior to the assessment. A modified version, BDI-II, has been developed and shown good psychometric properties in non-clinical adolescent samples (26). When this study was designed, the BDI-II was, however, not yet validated for the Swedish version and BDI-A1 is used instead. BDI consists of 21 items rated on a 4-point scale and yields a total score by summation of the ratings for the individual items.

Beck’s Anxiety Inventory (BAI) is often used in adolescent research and clinical practice, with good psychometric properties reported (27). BAI contains 21 items assessing the degree to which the respondent has been affected by the physical or cognitive symptoms of anxiety during the past week. BAI items are also meant to reflect panic attack symptoms.

Strengths and Difficulties Questionnaire (SDQ) is an internationally used screening instrument for mental health problems in children and teenagers (28). It is made up of 25 statements regarding psychological attributes and behaviours, forming five subscales: hyperactivity/inattention, emotional symptoms, conduct problems, peer problems and pro-social behaviours. All subscales except the pro-social scale are summarized to generate a total difficulties score. In this study, only the emotional scale (SDQ-em) was used. Acceptable psychometric properties for the self-report version of SDQ for adolescent have been shown in the previous studies and SDQ-em has been shown to have valid ability to differentiate cases of AD and MDD in this age group (29).

Physical activity was reported on a five point scale as frequency of exercising with hard breathing and sweating (never, seldom, once a week, twice a week, >twice a week).

### Blood chemistry and body mass index

Capillary blood samples were drawn right after the ECG registration and non-fasting p-glucose was analysed with a portable Heamocue Glucose System devise (30). Weight and length were measured and BMI calculated [BMI = weight (kg)/length (m^2^)].

### Statistical analyses

Variables with a positively skewed distribution were logarithmically transformed. Heart rate and logarithmically transformed HRV parameters were normally distributed in the clinical and controls sample. Groups were compared in a two tailed fashion, with the two sample *t*-test or, when normal distributions were absent, with Mann–Whitney’s *U*-test. Relations between psychiatric self-assessment and HRV indices were assessed by Pearson’s product-moment correlation, and when variables were of an ordinal nature, Spearman’s rank correlation was used. Partial correlations were used to remove the effect of heart rate (HR), BMI, systolic (SBP) and diastolic blood pressure (SBP), p-glucose and physical activity on HRV. Partial correlations are correlations of the residuals obtained from regressions where HR, BMI, SBP, DBP, p-glucose and physical activity have acted as predicting variables on the respective HRV indices. Such operations render the underlying correlations when the effects of the predicting variables have been removed. Linear multiple regressions with BMI, SBP, DBP, p-glucose and physical activity as co-variates were applied to all HRV-variables. The results from these two strategies did not, contradict each other, but served to further support the underlying theories.

Cohen’s *d* were calculated as effect sizes using the pooled standard deviations (*d*= m_1_– m_2_/√[(σ_1_^2^ + σ_2_^2^)/2]) (Cohen, 1988). A *d* of 0.20 represents a small effect size, 0.50 represents a medium effect size and 0.80 represents a large effect size. The three diagnostic subgroups of the clinical sample were analysed with one-way ANOVA and compared by contrast analysis. The ANOVA was executed by the method of variance components with diagnosis and SSRI used as random factors. This method has the advantage of revealing the relative influence of diagnosis and SSRI on the residual variation, when the effects of the fixed factors have been removed. The variables HR, BMI, SBP, DBP, p-glucose and physical activity served as fixed factors.

## Results

### Sample characteristics

**Figure 2:**
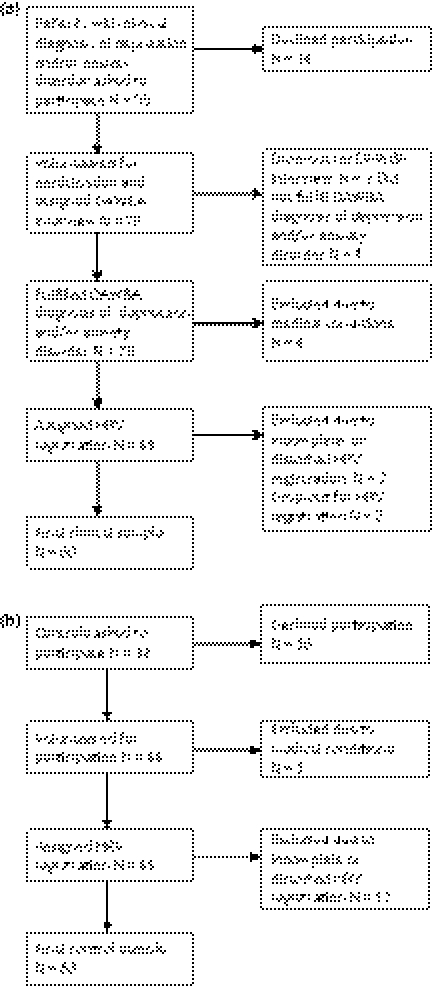
(a) Exclusion procedure of the clinical sample. (b) Exclusion procedure of the non-clinical sample.

From the original clinical sample (n = 73), one subject was excluded because of pregnancy and three because of somatic disorders (diabetes and hypothyreosis). Another seven patients were excluded as a result of >5% missing or distorted HRV data and two subjects dropped out. The final clinical sample included 60 subjects, (AD n = 20, MDD n = 11, comorbidity of AD and MDD n = 29). The clinical sample contained 23 patients with anti depressant medication of which 22 had SRRI medication (citalopram, fluoxetin and sertralin) and one had a tricyclic antidepressant (tryptizol). No patients were taking Seretonin Noradrenalin Reuptake Inhibitors. From the original control sample (n = 66) one subject was excluded as a result of thyrotoxicosis and twelve were excluded because of >5% missing or distorted HRV data, rendering a final control sample of 53 subjects (Fig. 2a,b).

Background factors such as parent unemployment or single parent household did not differ between the clinical sample and the controls. The control sample contained a higher ratio of subjects with parents of non-Swedish origin. Use of tobacco, SBP, DBP and self-reported physical activity showed no significant differences between the clinical sample and the controls, but BMI was lower and p-glucose higher in the clinical sample (Table 1). The difference of p-glucose between the samples was partly explained by four subjects in the clinical sample with high p-glucose (>12 mmol/L) but no decrease of HRV compared with the rest of the subjects in this sample (data not shown).

**Table 1:** Comparison of back ground characteristics in the clinical sample and in the controls

	Clinical sample n = 60^1^	Controls n = 53	
	N/mean (SD)	N/mean (SD)	X2/z-value/t-value
Parents’ occupational status (n = 58)^2^			
Both parents employed	44	39	0.96 ns
At least one parent unemployed	10	14	
Family situation (n = 56)^2^			
Living with two parents	39	38	0.06 ns
Living with single parent	17	15	
Parents’ ethnical background (n = 58)^2^			
At least one parent Swedish	55	44	4.01*
Both parents of foreign origin	3	9	
Daily smoking of cigarettes (n = 58)^2^			
No	49	46	
Yes	9	7	0.12 ns
Physical Activity Index (n = 58)	3.11	3.47	Z = 1.56 ns
Body Mass Index (n = 57)	20.8 (2.2)	22.3(3.7)	t = 2.63*
p-glucose (n = 59)	6.7 (2.2)	5.5 (0.7)	Z = −3.41**
Systolic blood pressure (n = 58)	109.7 (11.6)	111.4 (9.3)	t = 0.85 ns
Diastolic blood pressure (n = 57)	68.5 (9.7)	67.2 (7.5)	t = −0.76 ns
BDI (n = 53)	25.4 (12.0)	9.2 (8.6)	t = −8.1***
BAI (n = 53)	22.3 (10.9)	12.0 (9.0)	t = −5.5***
SDQ-em (n = 53)	7.2 (1.6)	3.5 (2.3)	Z = −7.3***

1 The clinical sample consisted of 60 subjects but each subject has not delivered full data sets.

2 Parents occupational, family situation, ethnicity, snuff and cigarette consumption are used only to describe the representativity of the samples and are not introduced as covariates in the calculations.

*** t- and z-values were significant at p < 0.001

** p < 0.01

* p < 0.05, ns = non-significant.

Linear regression models applied on HF, LF and SDNN in both samples showed significant contributions from physical activity and DBP only in the non-clinical sample. BMI, p-glucose and SBP showed no contributions in any of the samples (Table 2).

**Table 2:** Linear Regression Models showing the effect of BMI, p-glucose, SBP, DBP and physical activity on each HRV parameter (a) in the non-clinical sample (b) in the clinical sample

	HF	LF	SDNN
	Beta (CI)	Beta (CI)	Beta (CI)
(a) Non-clinical sample			
BMI	0.12 (−0.16 to 0.40) ns	−0.19 (−0.47 to 0.09) ns	−0.05 (−0.33 to 0.23) ns
p-glucose	−0.01 (−0.27 to 0.25) ns	0.00 (−0.27 to 0.26) ns	−0.06 (−0.32 to 0.21) ns
SBP	0.06 (−0.26 to 0.38) ns	0.23 (−0.09 to 0.55) ns	0.13 (−0.19 to 0.45) ns
DBP	−0.37 (−0.68 to −0.07)	−0.35 (−0.66 to −0.04)	−0.41 (−0.71 to 0.10)
Physical activity	0.35 (0.09 to 0.61)	0.30 (0.04 to 0.56)	0.31 (0.05 to 0.56)
(b) Clinical sample			
BMI	−0.03 (−0.32 to 0.27) ns	−0.25 (−0.53 to 0.03) ns	−0.22 (−0.50 to 0.07) ns
p-glucose	−0.05 (−0.36 to 0.26) ns	0.04 (−0.25 to 0.34) ns	0.07 (−0.24 to 0.37) ns
SBP	−0.12 (−0.49 to 0.26) ns	−0.11 (−0.48 to 0.25) ns	−0.10 (−0.47 to 0.27) ns
DBP	0.02 (−0.37 to 0.41) ns	0.14 (−0.23 to 0.51) ns	0.05 (−0.33 to 0.43) ns
Physical activity	−0.07 (−0.36 to 0.23) ns	−0.12 (−0.40 to 0.16) ns	−0.08 (−0.36 to 0.21) ns

### Comparison of HRV in the clinical sample vs. controls

The HF, LF and SDNN were significantly lower (medium effect sizes) in the clinical sample with diagnoses of AD and/or MDD compared with the controls. No difference of heart rate was found between the groups (Table 3).

**Table 3:** Differences of mean heart rate and logarithmically transformed HF, LF, SDNN of the clinical sample compared with the controls

	Clinical sample	Clinical sample M (SD) n = 60	Controls M (SD) n = 53	Mean difference X1−X2	Cohens *d*	t-value
Log HF	Total	5.46 (0.86)	5.94 (0.83)	−0.46	0.57	2.96**
	SSRI−	5.64 (0.88)		−0.30	0.35	1.61 ns
	SSRI+	5.22 (0.79)		−0.72	0.89	3.45**
Log LF	Total	5.48 (0.86)	5.94 (0.87)	−0.48	0.53	2.86**
	SSRI−	5.58 (0.93)		−0.36	0.40	1.86 ns
	SSRI+	5.36 (0.76)		−0.58	0.71	2.73**
Log SDNN	Total	3.88 (0.34)	4.08 (0.33)	−0.20	0.60	3.19**
	SSRI−	3.94 (0.36)		−0.14	0.41	1.93 ns
	SSRI+	3.81 (0.30)		−0.27	0.86	3.38**
Heart rate	Total	73.2 (9.63)	76.2 (9.48)	−2.8	0.31	1.66 ns
	SSRI−	72.9 (10.32)		−3.3	0.33	1.52 ns
	SSRI+	72.9 (8.46)		−3.3	0.37	1.41 ns

ns = non-significant.

Patients with SSRI medication (SSRI+) and without SSRI medication (SSRI−) are analysed separately.

** Significant at p < 0.01.

### Analyses comparing diagnostic subgroups of the clinical sample

No significant differences of HF, LF or SDNN were found between the diagnostic subgroups (AD, MDD and comorbidity of AD/MDD). Patients with AD had lower LF (t = 2.02*), MDD had lower LF (t = 2.22*) and SDNN (t = 2.60*) and comorbidity of AD/MDD had lower SDNN (t = 2.12*) vs. the controls, when controlled for BMI, p-glucose, systolic and diastolic blood pressure and physical activity (Table 4).

**Table 4:** The SSRI effect on the contrast estimates of HF, LF and SDNN in all patients and the diagnostic subgroups vs. the controls, when controlled for BMI, p-glucose, systolic and diastolic blood pressure and physical activity

	HF		LF		SDNN	
	Contrast estimate (CI)	t-value	Contrast estimate (CI)	t-value	Contrast estimate (CI)	t-value
Patients vs. controls	0.41 (0.05 to 0.77)	2.26*	0.51 (0.14 to 0.89)	2.73**	0.21 (0.07 to 0.36)	2.98**
Without SSRI	0.20 (−0.22 to 0.61)	0.94 ns	0.37 (−0.08 to 0.82)	1.63 ns	0.13 (−0.03 to 0.31)	1.62 ns
With SSRI	0.63 (0.20 to 1.07)	2.94**	0.62 (0.17 to 1.06)	2.77**	0.27 (0.11 to 0.44)	3.25**
AD vs. controls	0.44 (−0.04 to 0.92)	1.81 ns	0.51 (0.01 to 1.01)	2.02*	0.18 (−0.01 to 0.37)	1.87 ns
Without SSRI	0.25 (−0.29 to 0.79)	0.93 ns	0.35 (0.22 to 0.93)	1.22 ns	0.15 (−0.07 to 0.37)	1.37 ns
With SSRI	0.88 (0.08 to 1.69)	1.18*	0.88 (0.04 to 1.74)	2.07*	0.24 (−0.08 to 0.56)	1.48 ns
MDD vs. controls	0.36 (−0.24 to 0.96)	1.19 ns	0.70 (0.07 to 1.32)	2.22*	0.31 (0.07 to 0.55)	2.60*
Without SSRI	0.00 (0.74 to 0.75)	0.99 ns	0.60 (−0.19 to 1.38)	1.51 ns	0.21 (−0.09 to 0.50)	1.37 ns
With SSRI	0.86 (−0.02 to 1.75)	1.93 ns	0.84 (−0.09 to 1.77)	1.78 ns	0.46 (0.10 to 0.81)	2.57*
AD + MDD vs. controls	0.41 (−0.05 to 0.87)	1.78 ns	0.42 (−0.05 to 0.89)	1.77 ns	0.19 (0.01 to 0.37)	2.12*
Without SSRI	0.23 (−0.29 to 0.87)	0.32 ns	0.36 (−0.25 to 0.97)	1.18 ns	0.14 (0.09 to 0.37)	1.23 ns
With SSRI	0.50 (−0.10 to 1.04)	1.64 ns	0.44 (−0.16 to 1.04)	1.44 ns	0.23 (0.00 to 0.46)	2.00*

* Significant at p < 0.05

** Significant at p < 0.01.

### The effect of SSRI on HRV

Significant differences of HF, LF and SDNN in the clinical sample vs. controls as well as between the diagnostic subgroups (AD, MDD, AD + MDD) vs. controls were only seen in patients with SSRI medication. Medication with SSRI explained 15.5% of the total variance of HF, 3.0% of LF and 6.5% of SDNN between the clinical sample and the controls when adjusted for BMI, SBP, DBP, p-glucose, physical activity. The results remained similar when self-assessed symptom severity (BDI, BAI and SDQ-em) were introduced as covariates (graph 1, Table 4).

The subgroups of patients with SSRI medication (n = 23) vs. patients without SSRI medication (n = 37) did not have significantly different BDI or BAI scores, but SDQ-em showed higher scores in the group without medication (Mann–Whitney *U*-test: z-value = −2.0, p-value 0.04). No differences of the covariates (BMI, systolic and diastolic blood pressure, p-glucose, physical activity) were identified between the subgroups (data not shown).

### Correlations of HRV and symptoms of anxiety and/or depression

In the control sample, significant correlations between HF and depressive symptoms (BDI) were found (correlation coefficient = −0.28 p = 0.047) after adjustment for BMI, SBP; DBP and p-glucose. This correlation did not remain significant when physical activity was introduced as a covariate. In the control sample, physical activity correlated to HF (0.38**), LF (0.33*), SDNN (0.34*) (Table 5). No significant correlations were found between the HRV indices and symptoms of anxiety (BAI) in the controls.

**Table 5:** Correlations between BDI, BAI, SDQ-em and logarithmically transformed HF, LF, SDNN analysed in the controls

	HF	LF	SDNN
Partial correlations controlling for cardiovascular risk factors: BMI, p-glucose, systolic and diastolic blood pressure			
BDI	−0.28*	−0.14 ns	−0.22 ns
BAI	−0.22 ns	0.01 ns	−0.13 ns
SDQ-em	−0.26 ns	−0.12 ns	−0.18 ns
Partial correlations controlling for cardiovascular risk factors: BMI, p-glucose, systolic and diastolic blood pressure and physical activity			
BDI	−0.26 ns	−0.11 ns	−0.20 ns
BAI	−0.25 ns	0.00 ns	−0.14 ns
SDQ-em	−0.20 ns	−0.06 ns	−0.12 ns

ns = non-significant.

* Significant at p < 0.05.

## Discussion

The main finding of this study is that HRV (HF, LF and SDNN) was significantly reduced in a group of adolescent psychiatric female patients with AD and/or MDD compared with healthy controls. The differences were of medium effect sizes and were not explained by BMI, SBP and DBP or physical activity, but partly by the effect of SSRI medication. The present data failed to support that physiological hyperarausal is specific for AD or that the decreased HRV in depressed patients is explained by co-morbid anxiety.

P-glucose was higher in the clinical sample, and could have contributed to the difference of HRV between the groups, even though p-glucose showed no contribution to HRV in any of the samples. Physical activity and DBP contributed to HRV in the non-clinical sample but not in the clinical sample and there were no significant differences of physical activity or DBP between the two samples.

Medication with SSRI explained 15.5% of the total variance of HF, 3.0% of LF and 6.5% of SDNN. This is in line with earlier findings in adult samples where SRRI contributed to a decrease of the RSA and SDNN in patients with AD and MDD (14,15).

Our limited clinical sample size may explain the lack of HRV difference between the diagnostic subgroups. The control sample of this study did not represent a true population sample, which limits the inferential power. However, the representativity of the control sample was supported by comparison with a large population based study (females n = 542), which showed that low RSA was related to depressive symptoms but not to anxiety in adolescent females (16). This compares well with data from our control sample in which HF was related to depressive but not anxious symptoms. Our analysis added the information that this relationship not remained significant after adjustment for physical activity. Physical activity is known to be related to increase of HRV in healthy adolescents (19). Physical activity was not correlated to BDI and BAI-score in the controls in this study but patients with AD were more physically active compared with patients with MDD or comorbidity of AD/MDD (data not shown).

The HRV-registration method in 4 min episodes is similar to the method used in a population based study (16). Four minutes registration of HRV has been shown to have intra-individual stability over 6 months in this age group (19).

In conclusion, adolescent females with AD, MDD and comorbidity of AD/MDD show a decrease HRV compared with healthy controls, which is partly explained by the effect of SSRI. The findings do not support that the diagnoses of AD and MDD can be differentiated by physiological hyperarousal in late adolescent girls. This is in line with earlier population based studies of younger adolescents.

## Abbreviations

AD: anxiety disorders
ANOVA: analysis of variance
BAI: Beck’s anxiety inventory
BDI: Beck’s depression inventory
BMI: body mass index
DAWBA: development and wellbeing assessment
DBP: diastolic blood pressure
DSM IV: diagnostic and statistical manual of mental disorders, 4th version
ECG: electrocardiogram
HF: high frequency
HRV: heart rate variability
ICD-10: International classification of diseases – 10th version
LF: low frequency
MDD: major depressive disorder
NA: negative affect
PA: positive affect
RSA: respiratory sinus arrhythmia
SDNN: standard deviation of inter beat interval
SDQ-em: strengths and difficulties questionnaire – the emotional subscale
SBP: systolic blood pressure
SSRI: selective serotonin reuptake inhibitors
